# Unveiling the Role of Guanidinium for Enhanced Charge Extraction in Inverted Perovskite Solar Cells

**DOI:** 10.1021/acsenergylett.5c00469

**Published:** 2025-05-09

**Authors:** Weidong Xu, Ganghong Min, Felix Utama Kosasih, Yueyao Dong, Ziyuan Ge, Qichun Gu, Muzi Chen, Richard A. Pacalaj, Tong Wang, Thomas Webb, Tian Du, Marcello Righetto, Guanjie He, Mischa Hillenius, Elizabeth von Hauff, Giorgio Divitini, Caterina Ducati, Martyn A. McLachlan, Franco Cacialli, Saif A. Haque, Artem A. Bakulin, James R. Durrant, Chieh-Ting Lin, Samuel D. Stranks, Thomas J. Macdonald

**Affiliations:** a Department of Chemistry and Centre for Processable Electronics, 4615 Imperial College London, London W12 0BZ, U.K.; b Department of Chemical Engineering and Biotechnology, 2152 University of Cambridge, Cambridge, U.K. CB3 0AS; c Department of Electronic & Electrical Engineering, 4919 University College London, London WC1E 7JE, U.K.; d Department of Materials Science and Metallurgy, University of Cambridge, 27 Charles Babbage Road, Cambridge CB3 0FS, United Kingdom; e Department of Materials and Centre for Plastic Electronics, Imperial College London, London SW7 2AZ, U.K.; f 28314 Max-Planck-Institut für Kohlenforschung, Kaiser-Wilhelm-Platz 1, D-45470 Mülheim an der Ruhr, Germany; g Forschungszentrum Jülich GmbH, Helmholtz-Institute Erlangen-Nürnberg (HI ERN), High Throughput Methods in Photovoltaics, Immerwahrstraße 2, 91058 Erlangen, Germany; h Department of Physics and Astronomy, London Centre for Nanotechnology, University College London, London WC1E 6BT, U.K.; i Department of Physics, Clarendon Laboratory, 145276 University of Oxford, Oxford OX1 3PU, U.K.; j Christopher Ingold Laboratory, Department of Chemistry, University College London, London WC1H 0AJ, U.K.; k Department of Physics and Astronomy, 1190 VU Amsterdam, De Boelelaan 1081, 1081 HV Amsterdam, The Netherlands; l Fraunhofer Institute for Electron Beam and Plasma Technologies, Winterberg Str. 28, 01277 Dresden, Germany; m Faculty of Electrical and Computer Engineering, TU Dresden, Nöthnitzer Str, 01187 Dresden, Germany; n Electron Spectroscopy and Nanoscopy, 121451 Istituto Italiano di Tecnologia, via Morego 30, 16163 Genoa, Italy; o Department of Engineering, 18956 Free University of Bozen-Bolzano, Università 5, Bolzano I-39100, Italy; p Department of Chemical Engineering, 34916 National Chung Hsing University, No.145, Xingda Road, South District, Taichung 40227, Taiwan

## Abstract

The incorporation of guanidinium (Gua) cations has significantly enhanced the optoelectronic properties of various perovskite compositions. When combined with other A-site cations in perovskite solar cells (PSCs), Gua cations not only enhance the power conversion efficiency of the solar cells but often improve their overall stability. While most studies examining the impact of Gua focus on PSCs with the n-i-p (conventional) structure, fewer have investigated its effects on the mechanism and performance of the p-i-n (inverted) structure. We investigate how partially substituting A-site cations with Gua affects the performance of PSCs and the associated charge carrier dynamics. Enhanced performance is observed in Gua-substituted inverted PSCs, primarily due to improved short-circuit current density and fill factor values. Our spectroscopic and microscopic analyses reveal that these enhancements stem from accelerated charge transport within the perovskite layer combined with inhibited ion migration following Gua incorporation, attributed to the reduction of localized inhomogeneities, which also notably enhance device stability. Our findings elucidate the role of Gua in inverted PSCs, showing negligible impact on open-circuit voltage but significant improvement in charge extraction efficiency. This contrasts with previous reports on conventional structures, where performance enhancement is primarily attributed to trap state reduction, resulting in higher open-circuit voltage.

## Graphical abstract

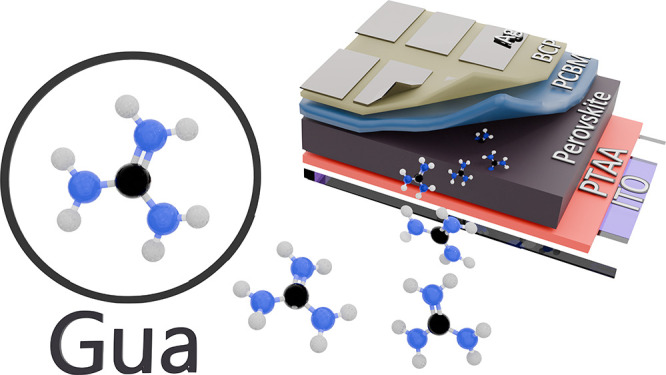

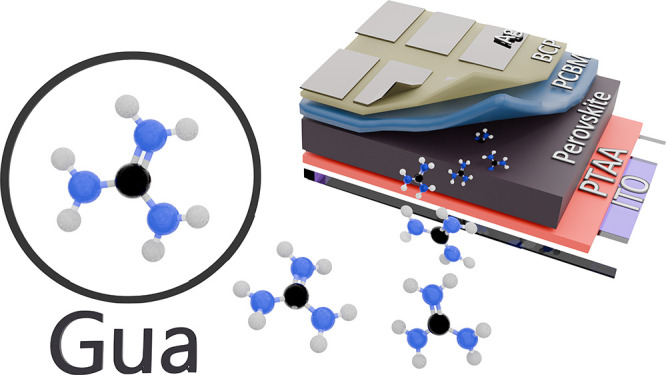

Organic–inorganic metal halide perovskite solar cells (PSCs) with an inverted (p-i-n) structure have shown comparable power conversion efficiency (PCE) but better long-term stability under light and heat stress compared to the conventional (n-i-p) structures.^1–5^ Moreover, the inverted structure is also attractive for compatibility with a wide range of perovskite-based tandem device architectures, with both practical and theoretical efficiency going beyond any single-junction cells.^6–11^

Enhancing perovskite film quality stands as a key strategy for achieving outstanding performance in solar cells. Over the past decade, various methods have been developed, encompassing precursor engineering, materials composition adjustments, deposition optimization, post-treatment techniques and surface passivation.^12–21^ Among these methods, the substitutional alloying of cations has emerged as a widely adopted approach for achieving highly efficient and stable PSCs where the substituted species aids the formation of a stabilized perovskite crystal structure and passivates undesirable trap states.^22–29^ A particularly noteworthy avenue involves studies demonstrating the substantial improvement in photovoltaic properties and device stability by replacing a small fraction of the A-site cation with guanidinium (Gua) or using Gua as an additive in conventional PSCs.^30–35^ It has been shown that incorporation of Gua into the structure of metal halide perovskites can result in a distortion of the crystal lattice which increases the activation energy for otherwise mobile iodide, thus improving the stability of the PSCs.^26,36,37^ Additional studies have also demonstrated improved performance, but this is typically only observed when a small amount of Gua is introduced due to its large ionic radius.^38–40^ The advantage of employing either partial substitution or additive use lies in their ability to streamline fabrication processes, reducing the need for post-treatment steps, and introducing minor electronic structural modifications without altering the device architecture or fabrication procedures. To date, numerous studies have investigated the benefits of cation substitution with Gua in the conventional structure, demonstrating improved open-circuit voltage and operational stability, mainly due to Gua’s suppression of the nonradiative recombination and ion migration in the perovskite layer.^26,37–39^ However, its application in the inverted structure has been less reported and less effective, achieving lower PCE compared to the conventional structure.^40–43^ The mechanism behind the enhancement in the inverted PSC performance requires further exploration.

To elucidate the influence of Gua incorporation on the performance of inverted PSCs, we focused on studying the substitution of a mole fraction (5%) of the A cation with Gua in methylammonium lead tri-iodide (MAPbI_3_) perovskite precursor solution and its corresponding devices. We propose 5% Gua as the optimal loading amount since it has been previously shown to have no major effect on the band gap and absorption (see Figure 1a).^36^ Exceeding this amount has been previously shown to reduce current density and fill factor in PSCs.^44^ To confirm this, Figure S1 shows our device optimization statistics which is consistent with observations in the literature supporting that a nominal 5% Gua is most suitable for our PSCs. Our focus on partial substitution, rather than additives, is aimed at maintaining the stoichiometry of the perovskite composition. This approach helps to avoid additional complexities introduced by excess cations at the surface or grain boundaries, which could lead to passivation or barrier formation.^16,45^ Our investigation delves into the structural, morphological, compositional and optoelectronic properties of the perovskite materials, exploring the impact of these properties on charge recombination, transport and extraction processes. Interestingly, our findings reveal a different possibility from the observed device performance in previous reports on the conventional structure.^26,31,39^ Specifically, we observed a preserved open-circuit voltage (*V*_*OC*_) alongside improved short-circuit current density (*J*_*SC*_) and fill factor (FF) values in the MA_1–*x*_Gua_*x*_PbI_3_ PSCs. Operando photoluminescence (PL) spectroscopy, time-resolved photoluminescence (TRPL) and optical-pump terahertz (THz)-probe spectroscopy were employed to gain insights into these observations.^46,47^ Our results suggest that the improved device performance in MA_1–*x*_Gua_*x*_PbI_3_ PSCs can be primarily attributed to fast and enhanced charge extraction. Furthermore, we note improved stability in the inverted MA_1–*x*_Gua_*x*_PbI_3_ PSCs is associated with the inhomogeneity of the perovskite layer. Finally, to indicate that the benefits of Gua were not limited to MAPbI_3_ PSCs, we fabricated formamidinium (FA)-based mixed-cation PSCs which demonstrated the same trends in improved performance. However, these mixed-cation PSCs contain two mixed A-site cations and two halides, which complicates our investigation on the role of substituted Gua. Thus, this manuscript is focused on unveiling the role of Gua within the simplest and most well-studied perovskite absorber layer, MAPbI_3_.

**Figure 1:**
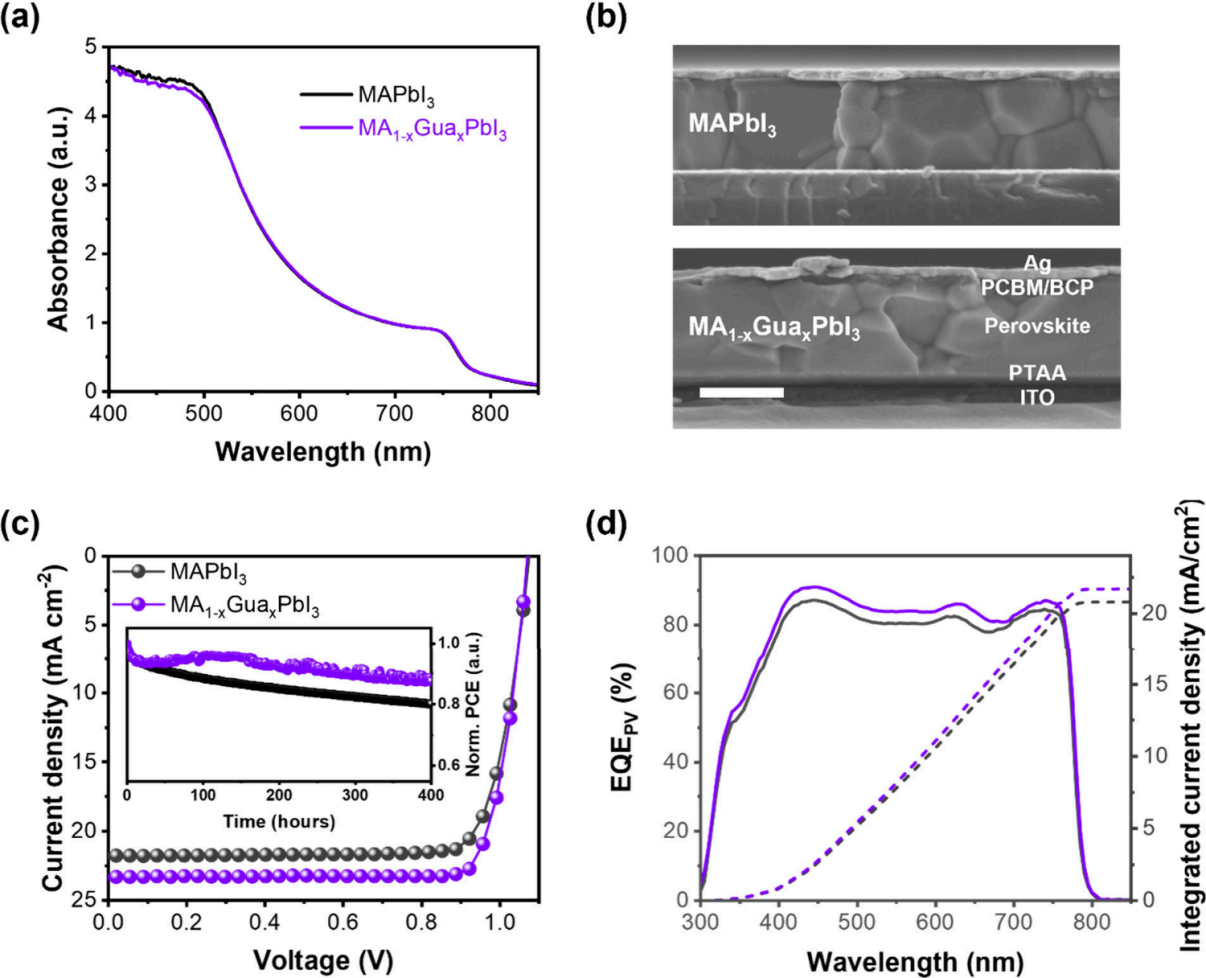
(a) UV–vis Absorption spectra of neat MAPbI_3_ and MA_1–*x*_Gua_*x*_PbI_3_ films. (b) Cross-section SEM images and device structure of MAPbI_3_ and MA_1–*x*_Gua_*x*_PbI_3_ PSCs with scale bar of 500 nm. (c) Device *J*–*V* and (d) external quantum efficiency characteristics of champion MAPbI_3_ (black) and MA_1–*x*_Gua_*x*_PbI_3_ (purple) PSCs. The inset figure in (c) shows stability test data of the two devices operated at MPP under continuous 1-sun-equivalent LED illumination.

We first fabricated the MA_1–*x*_Gua_*x*_PbI_3_ and MAPbI_3_ based perovskite solar cells and characterized their performance, as shown in Figure 1. Figure 1b shows the cross-section SEM images of the respective devices with the structure ITO/PTAA/PFN-Br/Perovskite/PCBM/BCP/Ag. For a detailed description of the device fabrication, please refer to the Supporting Information. Figure 1c reports the *J*–*V* characteristics of the champion devices, revealing a higher PCE of 20.92% for MA_1–*x*_Gua_*x*_PbI_3_, surpassing the PCE of 18.98% for MAPbI_3_. This is the highest reported value so far for a Gua substituted MAPbI_3_ PSC based on the inverted structure.^41,48,49^ This improvement in PCE is primarily attributed to enhancements in the short-circuit current (*J*_*SC*_) by 1.5 mA/cm^2^ and the fill factor (FF) from 0.81 to 0.84 upon incorporating Gua into the MAPbI_3_ system, while the open-circuit voltage (*V*_*OC*_) remains consistent, as indicated in Table S1. The statistical analysis of these parameters from 46 devices is further depicted in Figure S2 and summarized in Table S1, confirming the same behavior from the champion devices. Additionally, a higher external quantum efficiency (EQE) is evident in MA_1–*x*_Gua_*x*_PbI_3_ devices compared to MAPbI_3_, as illustrated in Figure 1d, aligning with the increased *J*_*SC*_ observed in the *J*–*V* measurements. Notably, the unsealed MA_1–*x*_Gua_*x*_PbI_3_ PSC exhibits enhanced operational stability, maintaining 90% of its original PCE in a 400-h maximum power point (MPP) test in a nitrogen filled glovebox, which is comparable with the best-performing MAPbI_3_ solar cells using other modification methods.^50–52^ In contrast, the MAPbI_3_ device only retained 85% of its original PCE during the same test (insert figure of Figure 1c). These results underscore a significant improvement in both photovoltaic performance and operational stability achieved by introducing small amount of Gua. Furthermore, we fabricated solar cells based on FA_0.97_MA_0.03_Pb­(I_0.97_Br_0.03_)_3_ using the same method, with 5% Gua substitution of FA cation in the precursor solution. Upon Gua incorporation, we observed performance enhancement, with a significantly higher PCE of 21.45% compared to 20.37% for the reference, attributed to improvements in both the *J*_*SC*_ and FF, as illustrated in Figure S3, further supporting the observation that devices incorporating Gua exhibited higher *J*_*SC*_ and FF overall compared to the reference cells. Notably, these devices were also passivated with phenethylammonium bromide, and the enhancement in performance mirrored the trend observed in the passivation-free methylammonium (MA) system, suggesting that this improvement may be extended across other perovskite systems.

To assess the influence of Gua incorporation on perovskite film characteristics, we examined the morphology and crystallinity of the perovskite films. Figure 1a and Figure 2a present the cross-sectional and top-view scanning electron microscopy (SEM) images of the MAPbI_3_ and MA_1–*x*_Gua_*x*_PbI_3_ films, respectively. Both fresh perovskite films exhibit similar morphological cluster sizes and a consistent film thickness of 600 nm. This morphological similarity is further confirmed by wide-field PL images, where both fresh MAPbI_3_ and MA_1–*x*_Gua_*x*_PbI_3_ films display comparable local PL intensity and submicrometric features (Figure 2b), though the MA_1–*x*_Gua_*x*_PbI_3_ film exhibits a more uniform PL distribution (see Figure S4 for distribution histogram). The crystallinity of the perovskite films was investigated using X-ray diffraction (XRD), with the results shown in Figure 2c. The XRD patterns for both films exhibit three main peaks, corresponding to (110), (220), and (310). However, in the case of the fresh films, a small PbI_2_ peak at 12.7° is evident only in the MAPbI_3_ (Figure S5a). A comparison of the reflection peaks for MAPbI_3_ and MA_1–*x*_Gua_*x*_PbI_3_ films reveals a slight peak shift toward lower angles in the MA_1–*x*_Gua_*x*_PbI_3_ film (Figure S5b), indicating a subtle variation in the unit cell. Further, Pawley refinement was conducted on the XRD patterns from Figure S5a to obtain accurate lattice parameters.^53^ The refined results summarized in Table S2 suggest an overall expansion of the unit cell volume from 1001.1 Å^3^ to 1003.7 Å^3^, with a minor decrease in the c parameter from 12.687 Å to 12.681 Å, and an elongation in the a parameter from 8.882 Å to 8.897 Å. This analysis suggests that Gua has been successfully incorporated into the MAPbI_3_ crystal lattice, causing no significant change in morphology, although a smaller crystallite size is observed in MA_1–*x*_Gua_*x*_PbI_3_ (181 nm) compared to MAPbI_3_ (204 nm) (fifth column, Table S2).^26,32,44^

**Figure 2:**
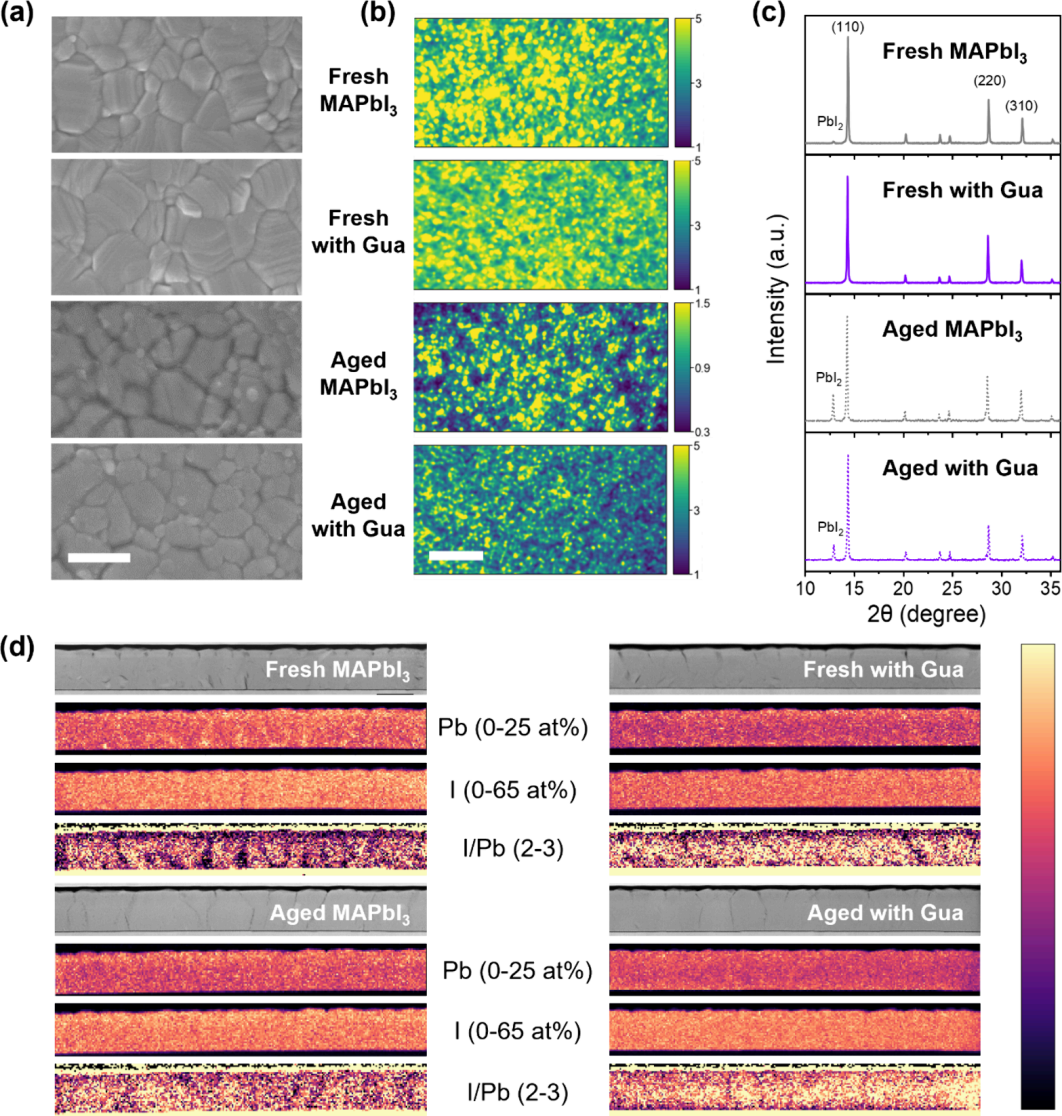
(a) SEM, (b) widefield PL images and (c) XRD of fresh and light-aged MAPbI_3_ and MA_1–*x*_Gua_*x*_PbI_3_ films, with top to bottom of fresh MAPbI_3_, fresh MA_1–*x*_Gua_*x*_PbI_3_, aged MAPbI_3_ and aged MA_1–*x*_Gua_*x*_PbI_3_. The scale bar in (a) and (b) represents 500 nm and 10 μm, respectively. (d) Cross-sectional STEM-HAADF images and STEM-EDX analysis of Pb, I and I/Pb ratio of MAPbI_3_ and MA_1–*x*_Gua_*x*_PbI_3_ devices with size of 5 × 0.7 μm for each individual image/map. The aging process was done under 1-sun-equivalent white-light LED illumination in N_2_, the aging time is 400 h for (a), (c), and (d) and 100 h for (b).

To understand how Gua improves the solar cell operation stability, we investigated the morphology, crystallinity, and their correlation to local PL intensity of perovskite films before and after aging under 1-sun-equivalent white-light LED illumination in N_2_. After the stability test, MAPbI_3_ films exhibit more erosion at their grain edges (Figure 2a), reduced homogeneity, and decreased PL intensity (Figure 2b and Figure S4) compared to MA_1–*x*_Gua_*x*_PbI_3_ films. This could be due to more severe photoinduced degradation of perovskite into PbI_2_, as illustrated in Figure 2c, where PbI_2_ peaks are more pronounced in the aged MAPbI_3_ films. The presence of PbI_2_, mostly formed at grain boundaries, could further accelerate degradation by introducing voids in the perovskite film through photolysis.^54,55^ The composition change is further evidenced by calculating the PL Centre of Mass (COM), representing the spectrally weighted average emission energy and extracted from the locally extracted PL spectra at each point in Figure 2b, as shown in Figure S6. This analysis allows one to compare the emission spectra change in their weight across the film.^56^ The COM maps demonstrate that there is minor spectral change in MA_1–*x*_Gua_*x*_PbI_3_ before and after aging, with more distinguishable submicrometric contrast and wider distribution (Figure S7) observed in MAPbI_3_. These morphologic and crystalline results suggest that the incorporation of Gua can inhibit photoinduced decomposition of perovskite, which could be attributed to suppression of submicrometric heterogeneity, most likely PbI_2_ clusters.

To gain deeper insights into the perovskite chemical composition distribution and investigate the reason behind the MA_1–*x*_Gua_*x*_PbI_3_ device’s better efficiency and greater stability, we employed cross-sectional high-angle annular dark field (HAADF) imaging and energy-dispersive X-ray spectroscopy (EDX) in a scanning transmission electron microscope (STEM) to study fresh and aged MAPbI_3_ and MA_1–*x*_Gua_*x*_PbI_3_ devices. The cross-sectional HAADF images of the four devices are shown in Figure S8. No significant differences in grain size or boundaries are observed between the fresh MAPbI_3_ and MA_1–*x*_Gua_*x*_PbI_3_ devices, corroborating the top-view SEM images shown in Figure 2a. Likewise, the grain distribution of aged MAPbI_3_ and MA_1–*x*_Gua_*x*_PbI_3_ perovskite are qualitatively similar. However, we observe slightly higher presence of more nonperovskite bright grains in both the fresh and aged MAPbI_3_ films compared to their MA_1–*x*_Gua_*x*_PbI_3_ counterparts (blue circles in Figure S8). Lead and iodine elemental maps and I/Pb ratio maps produced from the EDX analysis (Figure 2d) suggest that these bright grains are PbI_2_, as they correspond to low measured I/Pb ratios (∼2). The elemental and ratio maps also show that MA_1–*x*_Gua_*x*_PbI_3_ perovskite exhibits a more homogeneous I/Pb distribution both for the fresh and aged cases, whereas regions of nonstoichiometric I/Pb ratio are clearly observed for the MAPbI_3_ devices. These observations agree with the wide-field PL mapping results, where more submicrometric inhomogeneities are shown in both fresh and aged MAPbI_3_ films. These iodine-deficient regions are usually point defects, such as I vacancies, which will act as charge recombination centers, materials degradation centers or mediums for ion migration.^57–61^ These localized heterogeneities can also significantly affect photocarrier transport by neutral impurity scattering or Coulombic interaction from charged defects.^62,63^ Therefore, our STEM-EDX results indicate that one possible reason for the better efficiency and greater stability of the MA_1–*x*_Gua_*x*_PbI_3_ devices is the reduced point defects and spatial heterogeneity in the perovskite layer. This improvement may be attributed to the unique chemical properties of Gua, which has three NH_2_ groups compared to only one in MA, enabling stronger hydrogen bonding with I.^37^

To elucidate the mechanisms driving the enhancements in solar cell parameters and their correlation with observed chemical heterogeneities, we conducted operando PL studies on these devices. Operando PL measurement entails the simultaneous recording of absolute PL spectra while performing a *J*–*V* scan under 1-sun-equivalent illumination, as illustrated schematically in Figure 3a.^46^ It allows us to compare the real-time internal and external device performance over a range of voltage conditions by quantifying quasi-Fermi-level-splitting (*QFLS*), a value logarithmically proportional to the charge carrier density in the perovskite layer. The *QFLS* under open circuit (*QFLS*_*oc*_) determines the maximum *V*_*OC*_ level a device can achieve. Moreover, the *QFLS* at lower voltages (V ≤ *V*_*OC*_) indicates the densities of nonextracted photogenerated charges in the perovskite layer, which provides additional information about charge extraction efficiency. Figure 3b-c shows PL spectra with absolute photon radiance of the devices as the voltage is scanned from 0 to 1.2 V under 1-sun-equivalent continuous-wave (CW) laser illumination. By integrating the total emitted photons, we calculate radiative recombination current (*J*_*rad*_) and *QFLS* using the following equations:

1 $$QFLS=k_{B}T\cdot \ln\left(\frac{J_{rad}}{J_{0,rad}}\right)$$

where *J*_0,*rad*_ is the dark radiative recombination current density determined from the integral of the external quantum efficiency and the blackbody spectrum (Figure S9), *k*_*B*_ is Boltzmann’s constant and *T* is the temperature. Figure S10 illustrates the characteristics of the operando current and power densities under different voltages, with their parameters summarized in Table 1. These devices exhibit comparable performance to the champion devices (cf. Figure 1). Figure 3c shows the real-time *QFLS* in corresponding to the *J*–*V* is shown in Figure S10. Although the MAPbI_3_ devices exhibit slightly higher *V*_*OC*_ and *QFLS*_*oc*_, likely due to higher mobile ion densities (discussed further below), their impact on PCE is negligible compared to other parameters, as summarized in columns 2 and 3 of Table 1. Nevertheless, at low-voltage conditions (*V* ≤ MPP), consistently lower *QFLS* values are observed in the MA_1–*x*_Gua_*x*_PbI_3_ device, indicating more efficient charge extraction, agreeing well with the enhanced current densities demonstrated in Figure 1 and Table S1. We then compared the difference between *QFLS*_*oc*_ and *QFLS*_*sc*_, as summarized in column 7 of Table 1. The value of Δ*QFLS*_*OC‑SC*_ represents the effectiveness of charge extraction since it is exponentially proportional to the density of unextracted charge carriers remaining in the device. The MA_1–*x*_Gua_*x*_PbI_3_ device shows a value of 64 meV, meaning 91.6% of photogenerated charges were extracted. This is more efficient than the MAPbI_3_ device, whose 59 meV Δ*QFLS*_*OC‑SC*_ value corresponds to 89.8% charge extraction. The analysis of our operando PL data suggests that the device performance enhancement in MA_1–*x*_Gua_*x*_PbI_3_ is mainly attributed to its improved charge extraction efficiency rather than reduced nonradiative recombination velocities. Additionally, we investigated the temporal evolution of charge extraction in these devices under operational conditions using time-dependent PL measurements. Figure S11a depicts the schematic setups for this measurement, where continuous PL measurements were performed on complete devices held at short-circuit under 1-sun-equivalent CW laser illumination. Figure 3d illustrates the changes in PL intensity and current density over 30 min. We observed significant enhancement in short-circuit PL (*PL*_*SC*_) i.e. > 40% accompanied by a decrease in current densities of >1 mA in the MAPbI_3_ device. This indicates that an increasing number of charges accumulate in the perovskite layer, contributing to radiative recombination, while fewer charges are extracted. In contrast, MA_1–*x*_Gua_*x*_PbI_3_ devices exhibited negligible changes in both PL and *J*–*V* characters (Figure S11b-c), consistent with MPP results in Figure 1b, indicating better operational stability for MA_1–*x*_Gua_*x*_PbI_3_. Similarly, we observe that the neat MA_1–*x*_Gua_*x*_PbI_3_ film shows a moderate photobrightening effect, whereas the PL from MAPbI_3_ films increased to >40% within 30 min (see Figure S11d). These results suggest that the greater photostability of MA_1–*x*_Gua_*x*_PbI_3_ devices can mainly be attributed to their improved perovskite quality. Considering the observations from the STEM-EDX and PL mapping results, this can be attributed to the improved chemical homogeneity in MA_1–*x*_Gua_*x*_PbI_3_, while localized heterogeneities observed in MAPbI_3_ may lead to more severe ion migration or become centers of decomposition pathways.^64–67^ From our previous studies, we have noted that extra mobile ions, such as iodine vacancies likely present in MAPbI_3_, can screen the electric field, thereby enhancing charge accumulation at short-circuit conditions and reducing *J*_*SC*_.^46^ Meanwhile, mobile ions can boost *V*_*OC*_ due to reduced surface recombination, which explains the *V*_*OC*_ enhancement observed in Figure S11b.^46,68,69^ These results suggest that Gua incorporation may suppress ion migration, agreeing with many other reports.^36–38^ Likewise, we may also attribute the minor *V*_*OC*_ enhancement in the fresh MAPbI_3_ devices to the increased mobile ions densities.

**Figure 3:**
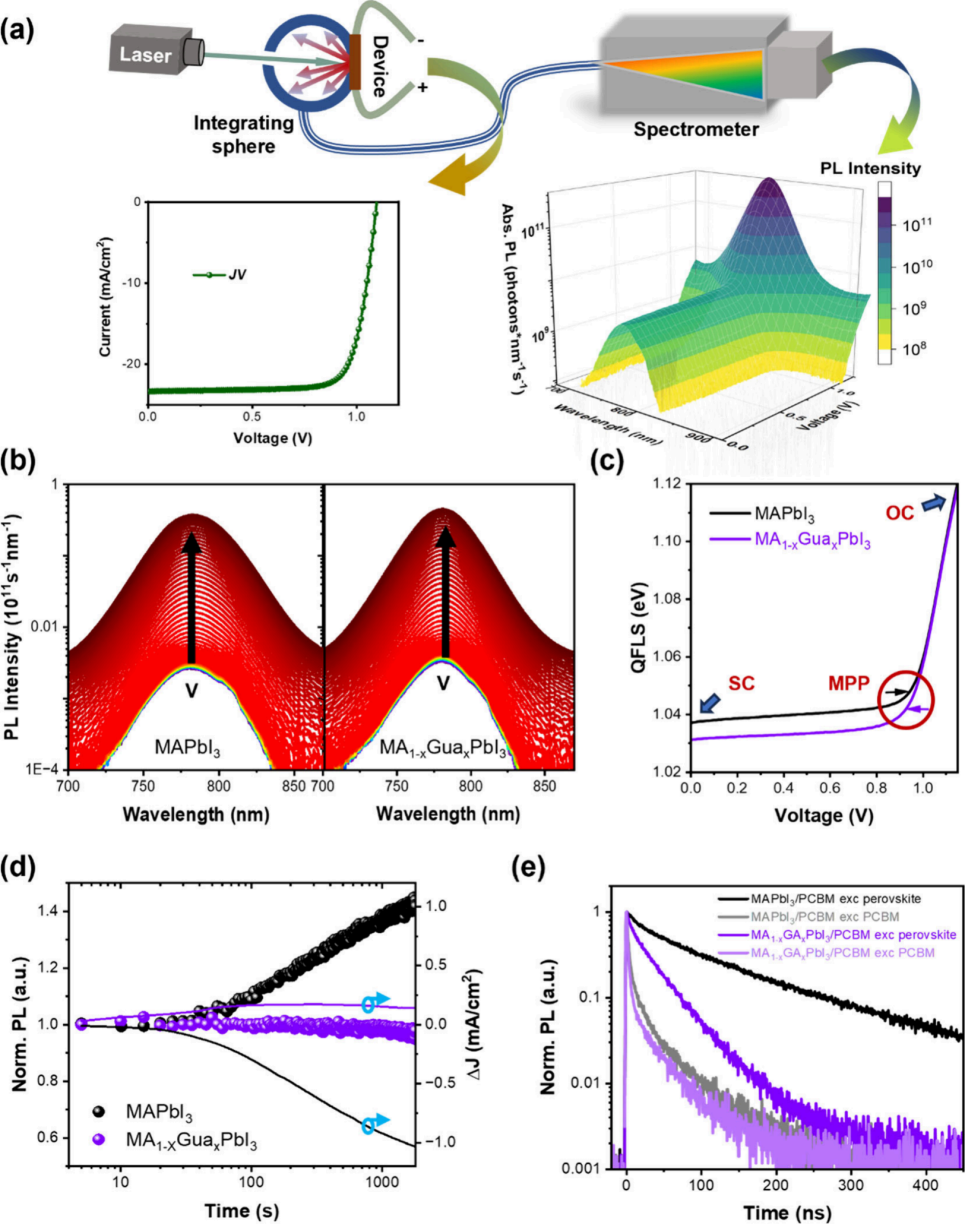
(a) Schematic drawing of operando PL set up. (b) Operando PL spectra of MAPbI_3_ and MA_1–*x*_Gua_*x*_PbI_3_ devices. The voltage increases from 0 to 1.2 V and is labeled with color change from blue to red. (c) Analysis of *QFLS* of MAPbI_3_ and MA_1–*x*_Gua_*x*_PbI_3_ devices under different voltages from operando PL measurements. MPPs are marked using small arrows. (d) Operando time-dependent PL measurement of MAPbI_3_ and MA_1–*x*_Gua_*x*_PbI_3_ devices at short-circuit. A 635 nm CW laser adjusted to 1-sun-equivalent intensity was used for excitation. (e) TRPL of perovskite/PCBM bilayers fabricated on glass substrates with front and back excitation with dark violet decay of MA_1–*x*_Gua_*x*_PbI_3_ excited from perovskite side, light violet decay of MA_1–*x*_Gua_*x*_PbI_3_ excited from PCBM side, black decay of MAPbI_3_ excited from perovskite side and gray decay of MAPbI_3_ excited from PCBM side. A 405 nm laser at frequency of 2 MHz and intensity of 10 nJ/cm^2^ per pulse was used for the measurement.

**Table 1:** Summary of Device Operando *QFLS* under Open Circuit, MPP, and Short Circuit Conditions of MAPbI_3_ and MA_1–*x*_Gua_*x*_PbI_3_ Solar Cells

Device	*V*_*OC*_ (V)	*QFLS*_*OC*_ (eV)	*J*_*SC*_ (mA/cm^2^)	*QFLS*_*SC*_ (eV)	PCE (%)	Δ*QFLS*_*OC‑SC*_ (meV)
MAPbI_3_	1.094	1.096 ± 0.001	22.37	1.037 ± 0.001	19.62	59
MA_1‑x_Gua_*x*_PbI_3_	1.093	1.095 ± 0.001	23.49	1.031 ± 0.001	20.48	64

To delve further into the origin of the improved charge extraction efficiency in fresh MA_1–*x*_Gua_*x*_PbI_3_ devices, we performed TRPL and THz measurements. Initially, TRPL was employed with spatially localized excitation and surface-quenching techniques to characterize the decay kinetics of charge transport layer (CTL)/perovskite films. This method allows us to differentiate between the kinetics of electron/hole transport within the perovskite layer and transfer across the CTL/perovskite interfaces, with or without electron/hole transport layers, as reported in our previous work.^47^ For this measurement, a 405 nm pulsed laser was used for excitation, resulting in photogenerated charge carriers localized to the perovskite surface at time zero due to its short penetration depth of approximately 30 nm in MAPbI_3_.^47^ We utilize both front and back excitation methods to selectively generate charge carriers at or away from the CTL/perovskite interface and use the CTL as a quencher for these charge carriers. This assumes that photogenerated charges will be transferred to CTL after reaching the CTL/perovskite interface. Therefore, this approach allows us to emphasize electron/hole transfer kinetics when charges are generated adjacent to the CTL/perovskite interface, and electron/hole transport kinetics when charges are generated on the opposite side.^47,70,71^ Figure 3e presents the decay kinetics of both MAPbI_3_ and MA_1–*x*_Gua_*x*_PbI_3_-based glass/perovskite/PCBM films. Interestingly, when excited from the PCBM side, the decay kinetics are nearly identical for both films, indicating similar electron transfer properties at the PCBM/perovskite interface. However, upon excitation from the glass side, the MA_1–*x*_Gua_*x*_PbI_3_ film exhibits much faster decay kinetics compared to MAPbI_3_. Since there is no significant difference between the front and back excitation decay kinetics in the glass/perovskite films (see Figure S12a), these faster kinetics suggest that electron transport within the MA_1–*x*_Gua_*x*_PbI_3_ layer is significantly enhanced compared to MAPbI_3_, as photogenerated free charges need to diffuse across the perovskite layer before transfer occurs at the PCBM/perovskite interface. Similarly, a reduced disparity is observed in the front and back excitation decay kinetics of ITO/PTAA/MA_1–*x*_Gua_*x*_PbI_3_ films, as depicted in Figure S12b. This suggests an enhancement in the hole transport property within the MA_1–*x*_Gua_*x*_PbI_3_ film. To quantify the improvement in charge carrier transport properties, we performed ultrafast optical-pump THz-probe measurements on neat perovskite films on deposited quartz substrates. A 400 nm pulsed pump beam is used to generate free charge carriers. The fluence excitation flux was kept low (13.2 μJ·cm^–2^) to avoid higher order relaxation processes, such as Auger and bimolecular recombination, on <100 ps time scale. Figure S13 illustrates optical-pump THz-probe dynamics of the MAPbI_3_ and MA_1–*x*_Gua_*x*_PbI_3_ films. The mobility of free charge carriers is calculated by Equation S1 in Supporting Information. The MA_1–*x*_Gua_*x*_PbI_3_ film exhibits a higher local mobility of 10.6 ± 0.06 cm^2^ V^–1^ s^–1^ compared to that of the MAPbI_3_ film which is 8.3 ± 0.03 cm^2^ V^–1^ s^–1^. The enhanced charge transport properties observed in the MA_1–*x*_Gua_*x*_PbI_3_ film, as evidenced by TRPL and THz measurements, play a crucial role in improving the overall charge extraction efficiency of the devices, contributing to enhanced FF and *J*_*SC*_. Taking account of the findings from XRD, STEM and EDX results, these improvements in charge transport properties of MA_1–*x*_Gua_*x*_PbI_3_ can be primarily attributed to its superior chemical homogeneity. Conversely, the presence of inhomogeneities observed in MAPbI_3_ may lead to charge scattering or the formation of barriers for charge transport, which impedes efficient charge collection toward the electrodes and hinders device performance.^47,72–74^

In summary, our study reveals a substantial enhancement in the efficiency and stability of inverted PSCs achieved through partial cation substitution of Gua. This enhancement is primarily attributed to the improved chemical homogeneity of the perovskite materials facilitated by Gua incorporation. This enhanced homogeneity results in better charge transport properties and reduced ion migration, thereby improving overall device charge extraction efficiency under low-bias conditions (*V* ≤ *V*_*MPP*_), leading to enhancements in both device *J*_*SC*_ and FF. Furthermore, this improved chemical homogeneity enhances the photostability of our perovskite films and extends device operational life. Our findings provide insights into the fundamental impact of Gua substitution on perovskite material properties, charge carrier dynamics, and device performance in inverted PSCs, offering a comprehensive understanding of Gua-modified perovskite applications.

## Supplementary Material



## Notes

Experimental section; additional *J*–*V* and device performance data; fitting of XRD data and calculation of lattice parameters and crystalline sizes; STEM-HAADF images; hyperspectral PL intensity and COM distribution histograms; *J*_0_rad_ calculation; operando *J*–*V*; operando time-dependent PL results; TRPL decays; time-dependent PL; optical-pump THz-probe dynamics and mobility calculation (PDF)
