# Supplementary material for: Unveiling the Role of Guanidinium for Enhanced Charge Extraction in Inverted Perovskite Solar Cells
Source: ACS Energy Lett. 2025 May 9;10(6):2660–9. doi: 10.1021/acsenergylett.5c00469 (PMC12172050; doi:10.1021/acsenergylett.5c00469)
Supplement: Supplementary file 1 [file nz5c00469_si_001.pdf]

Supporting Information

Unveiling the Role of Guanidinium for Enhanced Charge Extraction in Inverted Perovskite Solar Cells

Experiments

Materials preparation

Lead iodide (PbI_2, 99.99%), lead bromide ($PbBr_2$, 98%), [2-(3,6-Dimethoxy-9H-carbazol-9-yl)ethyl]phosphonic acid (Meo-2PACz, 98%) and methylamine hydrochloride (MACl, 98%) were from TCI. Guanidinium iodide (GuaI, 98%), Methylamine iodide (MAI), Methylamine bromide (MABr) and formamidinium iodide (FAI) were purchased from Greatcell Solar. Poly[bis(4-phenyl)(2,4,6-trimethylphenyl)amine (PTAA, 14,000 mW,) was purchased from Ossila. Poly(9,9-bis(3'-(N,N-dimethyl)-N-ethylammonium-propyl-2,7-fluorene)-alt-2,7-(9,9-dioctylfluorene))dibromide (PFN-Br) was from 1-Material. 1-Methyl-2-pyrrolidinone (NMP, anhydrous,99.5%), N, N-dimethyl formamide (DMF, anhydrous, 99.8%), Dimethyl sulfoxide, (DMSO, anhydrous, ≥99.9%), 2-propanol (IPA, anhydrous, 99.5%), methanol (MeOH, anhydrous,99.8%), and ethanol (EtOH, 99.8%) were from Sigma-Aldrich. Diethyl ether (anhydrous, 99.0%) was from JT Baker. All chemicals were used as received without any further purification.

Precursor solution preparation

1.5 M $CH_3NH_3PbI_3$ ($MAPbI_3$) precursor solution was prepared by dissolving PbI_2 and MAI at a molar ratio of 1:1 in anhydrous DMF:DMSO (9:1.1 volume ratio). For guanidinium (Gua) based $MAPbI_3$ precursor solution, MAI was replaced by GuaI in 5% and 15% molar ratio. 1.3 M formamidinium (FA) based perovskite ($FA_{0.97}MA_{0.03}Pb(I_{0.97}Br_{0.03})_3$) precursor solution was prepared by dissolving g 1.3 M ($FAPbI_3$) (dissolved in mixed solvents, DMF:NMP=8:2 vol.) and 1.3 M $CH_3NH_3PbBr_3$ ($MAPbBr_3$) (dissolved in mixed solvents, DMF:DMSO=8:2 vol.).

Solar cell fabrication

For the $MAPbI_3$ perovskite solar cells (PSCs) with and without GA: ITO was sequentially ultrasonically cleaned in acetone, Milli-Q water, acetone, and iso-propanol for 10 min in each solvent. The ITO was then dried with nitrogen and treated by oxygen plasma for 7 minutes. PTAA (2.5 mg mL^{-1} in toluene) was then spin-coated on the ITO at 5000 rpm (acceleration of 5000 rpm) for 20s. An ultrathin layer of PFN-Br (1-Material, 0.01 wt% in methanol) was then spin-coated on top of the

PTAA at 5000 rpm (acceleration of 5000 rpm) for 20s. $MAPbI_3$ precursor solution with or without Gua was spin-coated on the HTL at 4000 rpm (acceleration of 4000 rpm) for 20 s and after 7 s, 0.4 mL of diethyl ether was rapidly dropped on top of the spinning substrate. The substrate was then immediately annealed at 65 °C for 2 min before further annealing at 100 °C for 60 min. After annealing, all films were glassy black and allowed to cool for 10 min before deposition of the ETL. PCBM (Solenne 99.5% purity, 30 mg mL^{-1} in chlorobenzene) was then spin-coated on top of the perovskite layer at 2000 rpm (acceleration of 4000 rpm) for 20 s. BCP (Lumtec 99.5% purity, 0.5 mg mL^{-1} in methanol) was then spin-coated on top of the PCBM layer at 5000 rpm (acceleration of 4000 rpm) for 20 s. Immediately after BCP was deposited, the substrates were transferred (under nitrogen) to another glovebox and subject to thermal evaporation. Finally, 100 nm of Ag was thermally evaporated as a top contact at a base pressure of 5×10^{-6} mbar. For stability test at maximum power point (MPP) tracking, 3 nm of Cr and 100 nm Au (Cr/Au) were thermally evaporated instead of Ag at the same base pressure.

For the mixed cation-based PSCs: The glass-ITO substrates were ultrasonically washed with deionized water, acetone, and isopropanol for 10 minutes in sequence. After drying by N_2, the cleaned substrates were treated with ultraviolet ozone for 1 hour. A self-assembled monolayer (SAM) was deposited by spin-coating 1 mM Meo-2PACz in methanol at 3000 rpm for 20 seconds with 10 seconds dwell time before the spin-coating process. Then the ITO/SAMs was placed on a 100°C hot plate for 10 minutes. For the device with a PFN-Br layer, the PFN-Br solution (0.05 wt% PFN-Br in methanol) was spin-coated onto the ITO/SAMs at 4000 rpm for 220 seconds. Then, 1.3M mixed cation-based perovskite precursor solution with or without Gua was spin-coated onto the substrates at 4000 rpm for 20 seconds. At 10 second of the spin coating process, 0.4 ml diethyl ether was dripped onto the substrate. After spinning, MACl (solution in IPA) was coated at 4000 rpm for 20s. Both as-deposited perovskite thin films were heated at 60°C for 5 min and then placed on 100°C hot plate for 1 hour. The device was then completed with C60 (40nm, as ETL), BCP (5nm), and Ag (100nm) through thermal evaporation.

Solar Cell Characterization

J–V measurements were carried out under 1-sun (AM 1.5G) illumination using a calibrated solar simulator with a Xenon lamp (LOT). Device performance was measured with a Keithley 2400 source meter by scanning at 50 mV s^{-1}. Devices were fabricated using active areas of 0.045 and 0.09 cm^2.

External quantum efficiency (EQE) measurements were carried out with a halogen lamp chopped to a frequency of 188 Hz through a Newport monochromator and a 4-point probe in connection with a lock-in amplifier was used to collect data. The monochromatic beam was calibrated using a silicon photodiode and the data was analyzed with Tracer 3.2 software (LOT) to produce the EQE spectra.

Stability test of unencapsulated PSCs was carried out under continuous maximum power point (MPP) tracking mode and under open circuit with periodical J-V scan, both in a N_2 filled chamber (25 °C). Additionally, MPP tracking mode was carried out in ambient air (25 °C, 30 % humidity). In all cases, the PSCs are illuminated with white-light LED, calibrated to 1 sun condition by matching the measured J_{SC} with the value measured from AM 1.5 solar simulator.

Spectroscopic characterization

All devices and samples used for spectroscopic characterization were encapsulated in a nitrogen-filled glove box to prevent air exposure unless stated specifically.

Operando Photoluminescence Measurement: For PL spectrum collection, we employed a home-built system integrated with an AvaSphere-50-REFL integrating sphere (AVANTES), a Kymera 193i spectrograph (Andor), and a DU420A-BEX2-DD CCD camera (Andor). To apply voltage, record current, and facilitate photoexcitation, a potentiostat (Ivium Vertex.100 mA.EIS) and a CW 532 nm laser diode module (THORLABS, CPS532b) were utilized, respectively. System control and data collection were managed by a Labview code specially developed for this purpose. A mercury light calibration source (AvaLight-CAL-MINI, AVANTES) corrected the spectrometer wavelength, and an AvaLight-HALCAL-ISP50-MINI halogen light source (AVANTES) facilitated absolute photon flux

calibration. Laser excitation intensity was adjusted to 1-sun equivalent by aligning the J_{SC} of a PCBM device under a 532 nm laser with that under a solar simulator. During measurements, a mask smaller than the pixel area ensured complete coverage of all exposure areas with electrodes. Operando PL measurements were conducted at a scan rate of 7 mV s^{-1}. PSCs were soaked for 5 minutes under laser light at a bias voltage of 0.2 V, the default setting of the equipment, prior to measurement. A 5-minute wait time was observed as sufficient for device performance stabilization.

Time-Resolved Photoluminescence (TRPL) decays were recorded using the FLS1000 photoluminescence spectrometer from Edinburgh Instruments. Pulsed laser diodes emitting at a wavelength of 405 nm served as the excitation light source. The fluences were measured using an LED power meter, registering 6 mW/cm^2 (3 nJ/cm^2) for 405 nm at a frequency of 2 MHz. Photoluminescence signals were captured through a 700 nm long pass filter with a peak wavelength at 780 nm.

Operando time-dependent photoluminescence measurements were performed using a Horiba FL 1039 spectrometer with the set-ups shown in Figure S11a. During the measurement, each PSC was held at short-circuit condition and illuminated with a continuous-wave laser at an excitation wavelength of 635 nm, with intensity calibrated to 1 sun by matching the short-circuit current of the best perovskite solar cell under laser illumination to that under AM1.5 illumination. The short-circuit current at different time points was recorded using a power meter Keithley 2200.

Steady-state PL measurements were conducted with a Horiba FL 1039 spectrometer, illuminated with continuous-wave laser with excitation wavelength of 550 nm, step-size of 1 nm and integrating time of 1 s.

Ultraviolet-visible (UV-Vis) absorption spectra were measured with a Horriba UV-vis spectrophotometer by measuring both transmittance and reflectance spectra of the perovskite films, with step-size of 1 nm and integrating time of 0.5 s.

Optical-pump terahertz (THz)-probe spectroscopy:

The fundamental 800 nm laser beam (Astrella, Coherent, 4 kHz, ~35 fs) was divided into three portions, serving as the sources for the 400 nm pump beam, the THz probe beam, and the 800 nm gating beam. The 400 nm pump beam was generated via second harmonic generation using a beta-barium borate (BBO) crystal and modulated at 1 kHz using a chopper. It was directed onto a mechanical delay stage before converging with the probe beam, allowing precise control of the delay time between the pump and probe pulses.

The THz pulse was produced by irradiating a biased photoconductive low-temperature-grown gallium arsenide antenna (Tera-SED) with another portion of the 800 nm fundamental beam, modulated at 2 kHz. The remaining portion, maintained at 4 kHz, served as the gating beam, which interacted with the THz pulse transmitted through the sample in a collinear configuration with a ZnTe crystal. The electric field of the THz beam induced polarization change in the gating beam within the ZnTe crystal. The gating beam transmits through a quarter-wave plate and a Wollaston prism which divides the gating beam into two components which polarizations are orthogonal with each other. The orthogonal polarization components of the gating beam were then directed to two identical photodiodes. In the absence of the THz beam, the individual signals detected from these two components are balanced by adjusting the angle of the quarter-wave plate. When the THz beam is present, a differential voltage is generated between the two photodiodes and processed by a lock-in amplifier (Zurich MFLI), which is proportional to the electric field strength of the THz pulse.

The carrier mobility (μ) is calculated by[1]

$$\varphi\mu = -\epsilon_0 c(n_A + n_B)\frac{A_{eff}hc}{Ee\lambda(1 - T_{pump})}\left(\frac{\Delta E}{E}\right) \qquad Equation\ S1$$

where φ is unity assuming only free carriers are generated after pump excitation, ϵ_0 is the vacuum permittivity, c is the speed of light, e is the elementary charge, n_A and n_B are the refractive indexes of vacuum and the z-cut quartz substrate respectively, E is the 400 nm photon energy, T_{pump} is the transmittance of the sample at 400 nm, which is from a home-built UV-Vis absorption spectrometer. h

is the Planck constant, E is the electric field strength of the THz pulse transmitting through the sample without excitation, ΔE is the electric field strength change of the THz pulse following the 400 nm excitation, and A_{eff} is the effective overlapping area of the beams of the 400 nm pump and the THz probe. In this experiment, the beam size of the THz probe at the focus point is bigger than the 400 nm pump. Therefore, the effective area is approximately equal to the 400 nm pump beam size at the focus point, which equals to 3×10^{-7} m^{-2}.

Wide field hyperspectral PL measurements were carried out using a Photon etc. IMA system. For all measurements, ×100 air, chromatic aberration corrected objective lenses from Olympus (MPLFN and MPLAPON) were used. All samples were stored in a nitrogen filled glovebox until immediately before measurement to mitigate oxygen and humidity related transient behaviour. A 405-nm continuous wave laser with intensity of 80 mW/cm^2 was used for luminescence excitation, which travels through the objective to the sample. The emitted PL from the sample was incident on a volume Bragg grating, which splits the light spectrally onto a CCD camera. The detector was a 1,040×1,392 resolution silicon CCD camera kept at 0 °C with a thermoelectric cooler and has an operational wavelength range of 400–1,000 nm. By scanning the angle of the grating relative to the incident light, the spectrum of light coming from each point on the sample could be obtained. The system is calibrated by using a white light lamp from Ocean Optics with known spectrum and irradiance.

Scanning electron microscopy (SEM) measurements

SEM images were obtained using a LEO Gemini 1525 field emission gun scanning electron microscope. The acceleration voltage was set at 3-5 kV. All films were coated with a thin chromium layer.

X-ray diffraction (XRD) characterization.

X-ray diffraction (XRD) patterns were obtained using a Bruker D2 PHASER diffractometer Cu Kα (λ =1.5406 Å) source, samples were spun during measurement.

Scanning transmission electron microscopy (STEM)

For (S)TEM characterization, cross-sectional lamellae were prepared with an FEI Helios Nanolab Dualbeam FIB/SEM following a standard procedure described elsewhere.[2] The lamellae were immediately transferred into an FEI Tecnai Osiris (S)TEM, minimizing air exposure to ~2 min. This instrument was operated with a 200 kV beam. STEM-HAADF images were acquired using a Fischione detector, with a beam current of ~140 pA and a dwell time of 1 µs/pixel. STEM-EDX data was obtained using a Bruker Super-X silicon drift detector system with a collection solid angle of ~0.9 sr, a beam current of ~140 pA, a dwell time of 30 ms/pixel, a spatial sampling of 10 nm/pixel, and a spectral resolution of 5 eV/channel. The electron dose for STEM-EDX was ~2620 e-/Å^2, a value previously optimised with respect to beam-induced specimen damage and EDX data quality.[2] STEM-EDX data was processed in HyperSpy, an open-source Python package for multidimensional data analysis.[3] First, the EDX data was spectrally rebinned to 20 eV/channel, then denoised using principal component analysis to increase the signal-to-noise ratio.[4,5] Subsequently, the background-corrected intensities of X-ray peaks of interest were extracted. To obtain quantitative elemental maps, Cliff-Lorimer quantification was performed in each pixel using the X-ray peak intensity values.[6]

Supplementary Tables

Table S1. Summary of champion and statistical (23 devices) device performance of $MAPbI_3$ and 5% Gua-incorporated $MAPbI_3$ ($MA_{1-x}Gua_xPbI_3$) solar cells.

Device	J_{SC} (mA/cm^2)	V_{OC} (V)	FF	PCE (%)
$MAPbI_3$	21.80	1.076	0.809	18.98
Statistical	21.71 (± 0.62)	1.074 (± 0.004)	0.787 ((± 0.015)	18.36 (± 0.46)
$MA_{1-x}Gua_xPbI_3$	23.30	1.072	0.839	20.92
Statistical	22.86 (± 0.74)	1.070 (± 0.004)	0.804 (± 0.015)	19.65 (± 0.52)

Table S2. Lattice parameters and crystallite size calculated from XRD data from Figure 2b.

Sample	a (Å)	c (Å)	V (Å^3)	Crystallite size (nm)
$MAPbI_3$	8.8830 (± 5)	12.687 (± 2)	1001.1 (± 2)	204 (± 17)
$MA_{1-x}Gua_xPbI_3$	8.8967 (± 4)	12.681 (± 2)	1003.7 (± 2)	181 (± 12)

Supplementary Figures

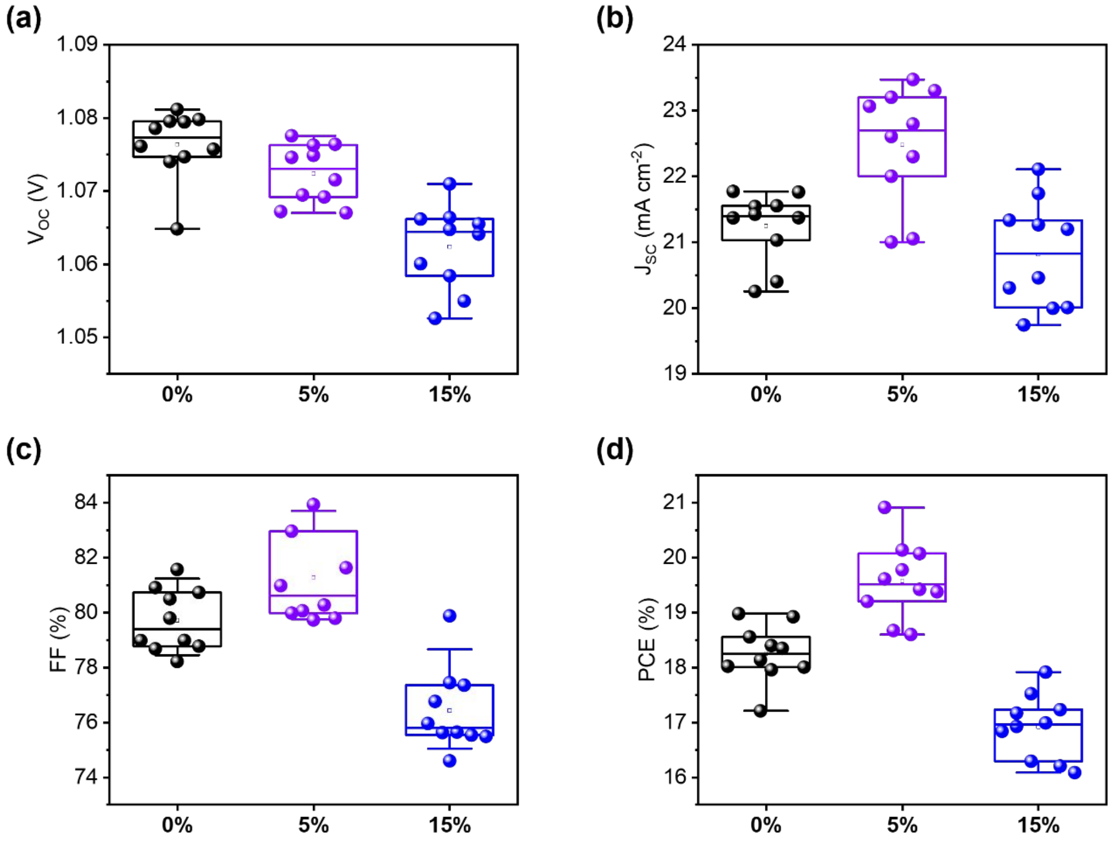

Figure S1. Performance statistics of optimized $MAPbI_3$-based PSCs with 0%, 5% and 15% molar ratio GuaI replacement of MAI in the precursor solution.

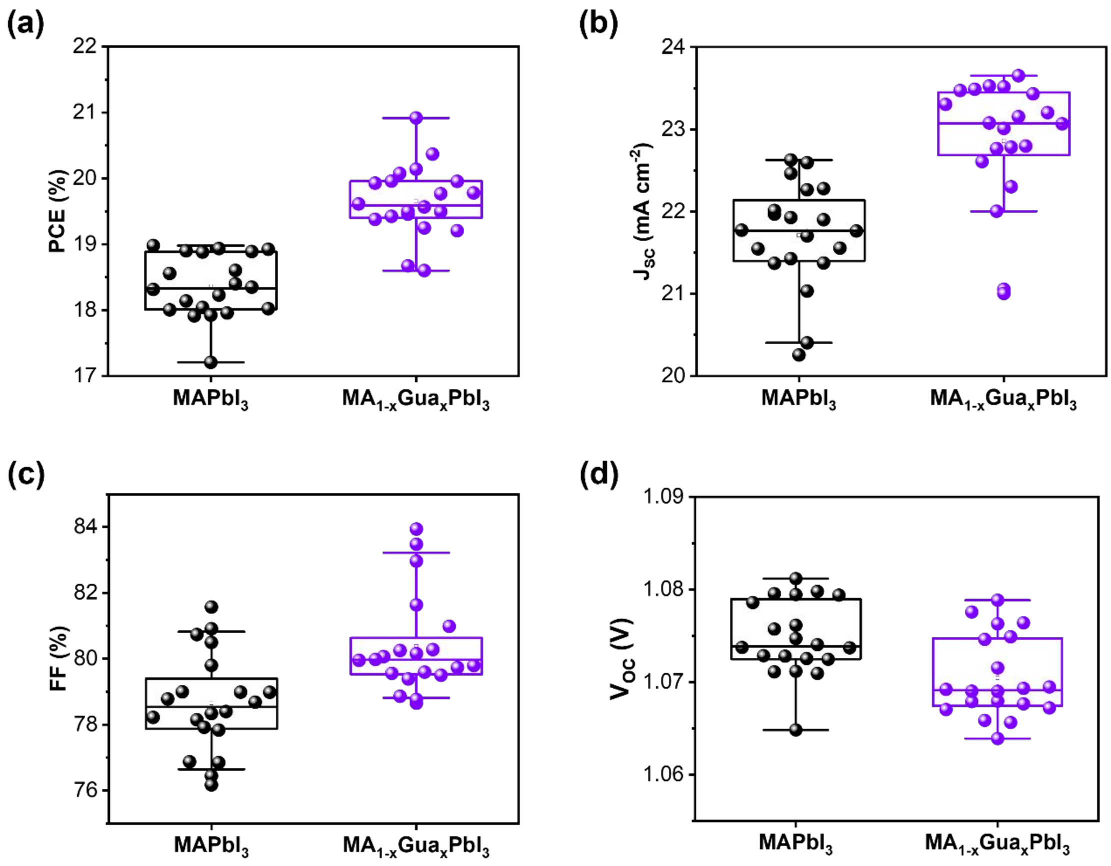

Figure S2. Statistics of (a) PCE, (b) J_{SC}, (c) FF and (d) V_{OC} from 23 $MAPbI_3$ and 23 $MA_{1-x}Gua_xPbI_3$ devices.

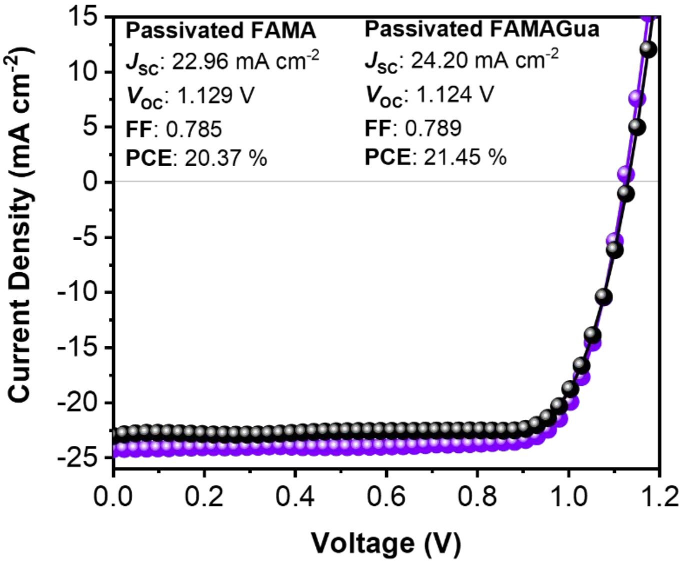

Figure S3. J-V characters and V_{OC}, J_{SC}, FF, PCE of mixed cation ($FA_{0.97}MA_{0.03}Pb(I_{0.97}Br_{0.03})_3$) PSCs with and without Gua incorporation.

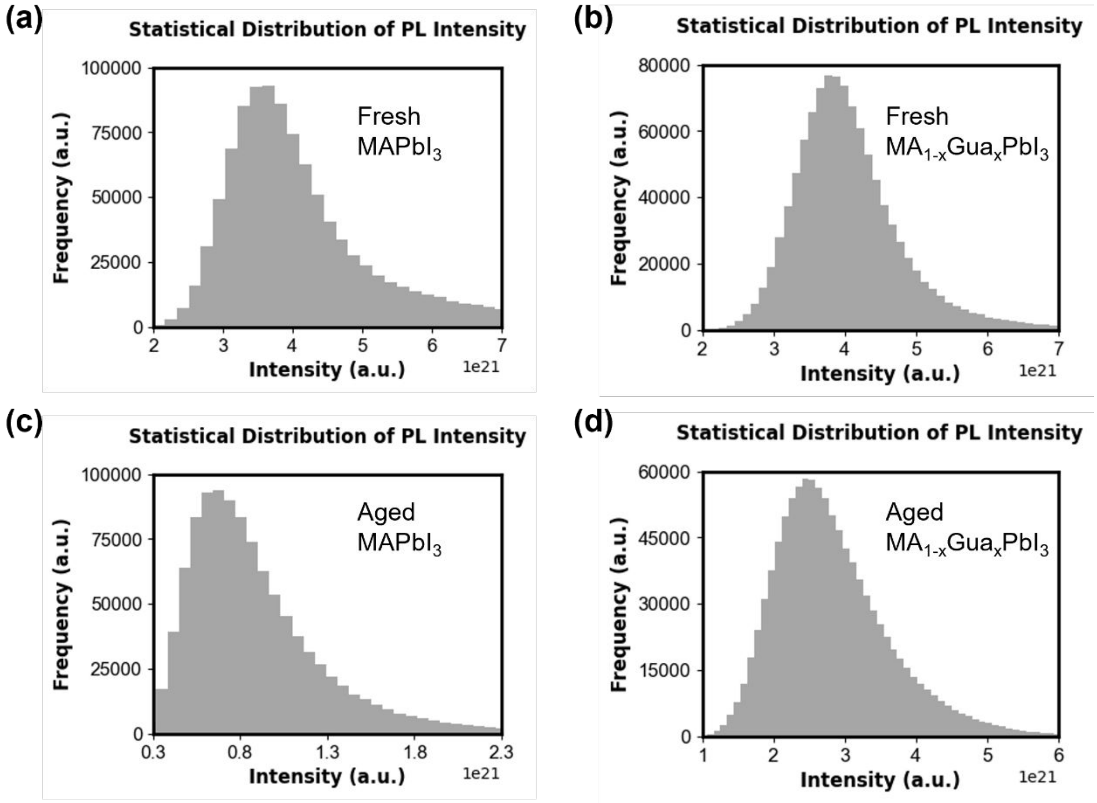

Figure S4. PL intensity distribution histograms for (a) fresh $MAPbI_3$, (b) fresh $MA_{1-x}Gua_xPbI_3$, (c) aged $MAPbI_3$, and (d) aged $MA_{1-x}Gua_xPbI_3$, derived from Figure 1b.

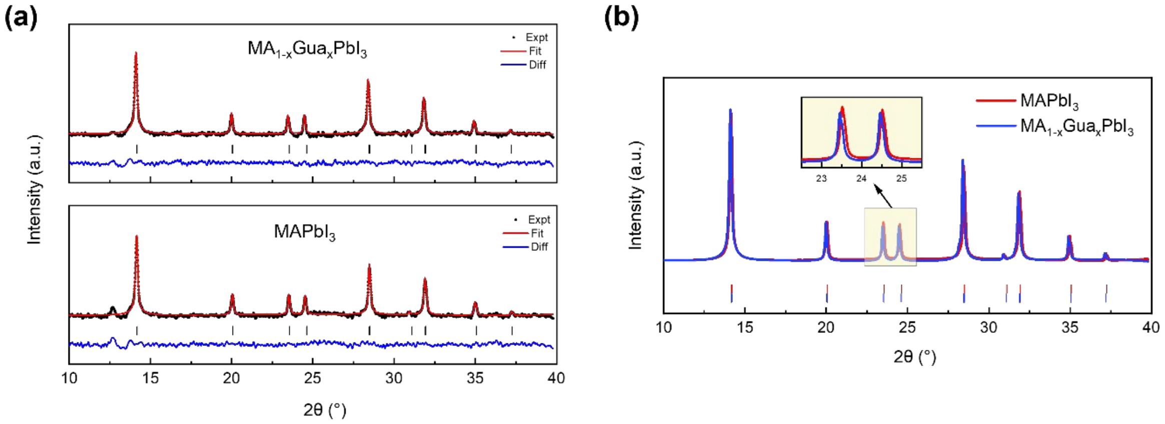

Figure S5. (a) XRD patterns of $MA_{1-x}Gua_xPbI_3$ and $MAPbI_3$ thin films. They are fitted using Pawley refinement.[7] The experimental data are displayed in black, the fitted data are in red, the difference between the two curves are in blue, and the permitted reflections are denoted by vertical bars. (b) Direct comparison of the fitted XRD data with zoomed window showing a slight peak shift towards lower angles in the $MA_{1-x}Gua_xPbI_3$ film.

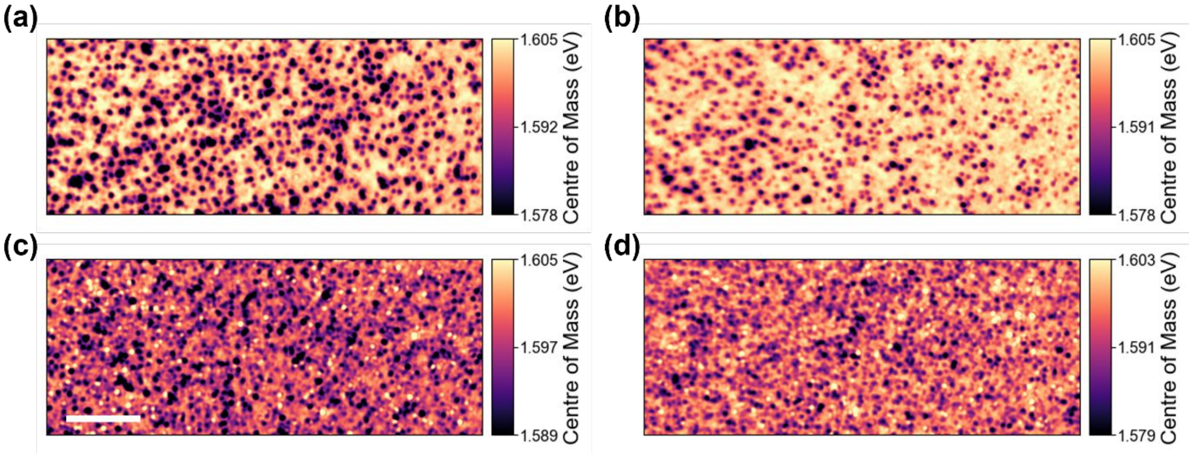

Figure S6. Centre of Mass (COM) mapping of (a) fresh $MAPbI_3$, (b) fresh $MA_{1-x}Gua_xPbI_3$, (c) aged $MAPbI_3$ and (d) aged $MA_{1-x}Gua_xPbI_3$ film. Calculated from the hyperspectral PL spectra from Figure 2b by interpolating the data along a uniform energy axis: $COM = \frac{\sum E_n \cdot I_{PL}(E)}{\sum I_{PL}(E)}$

Where I_{PL} is absolute intensity of photoluminescence, E is energy.

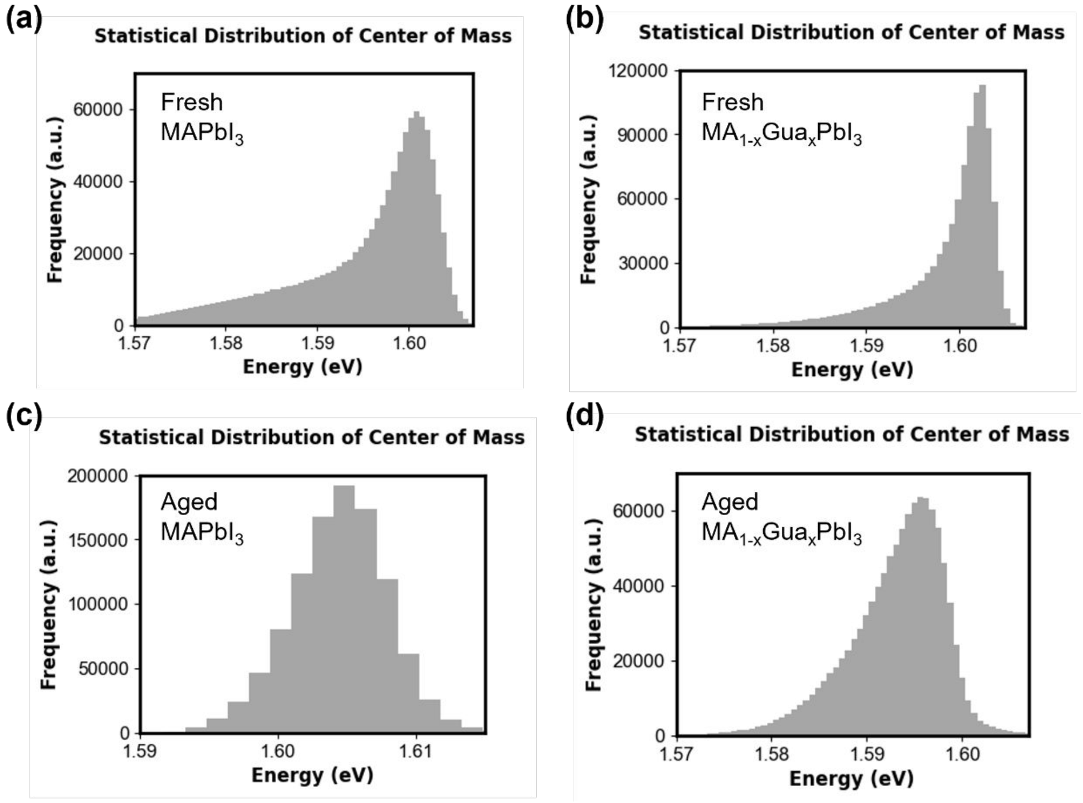

Figure S7. PL COM distribution histograms for (a) fresh $MAPbI_3$, (b) fresh $MA_{1-x}Gua_xPbI_3$, (c) aged $MAPbI_3$, and (d) aged $MA_{1-x}Gua_xPbI_3$, derived from Figure S4.

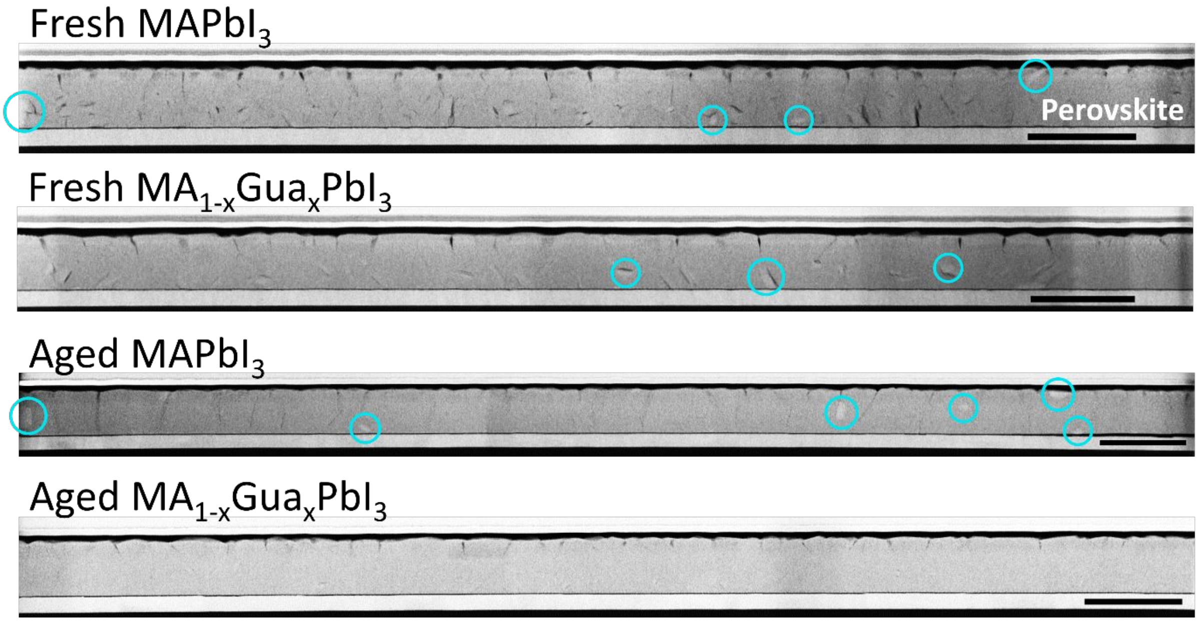

Figure S8. Cross-sectional STEM-HAADF images of fresh and aged $MAPbI_3$ and $MA_{1-x}Gua_xPbI_3$ devices. Blue circles mark non-perovskite grains, which EDX analysis suggests are PbI_2 (see Figure 2d in the main text). Scale bars indicate 1 μm.

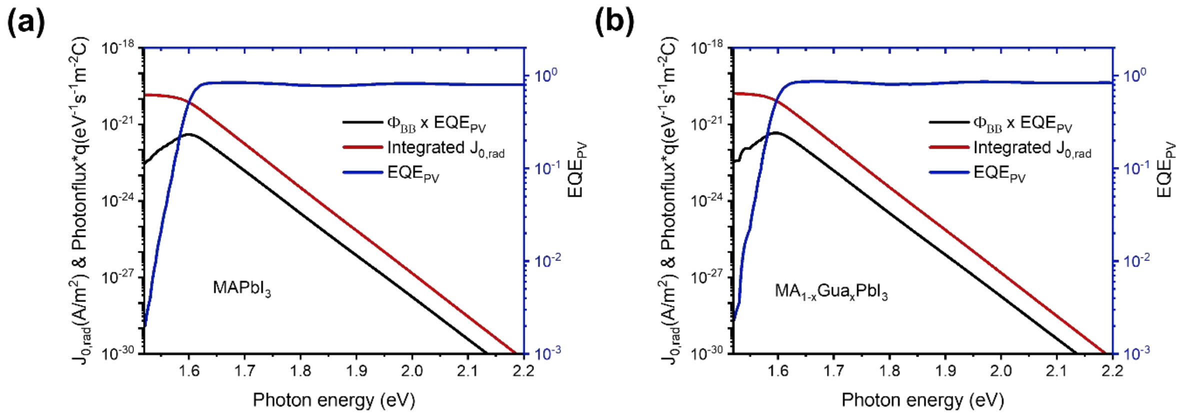

Figure S9. J_{0_rad} calculation of $MAPbI_3$ and $MA_{1-x}Gua_xPbI_3$ devices from EQE measurement.

EQE onset of the $MAPbI_3$ and $MA_{1-x}Gua_xPbI_3$ solar cells and their emitted spectral photon flux calculated when the devices are in equilibrium with the black-body (BB) radiation of the surroundings at 298 K.

The QFLS was determined using Equation 1 and follows an approach reported by Rau et al,[8] where J_{rad} was calculated by converting the absolute emitted photon numbers into radiative currents (per photon per electron-hole pair). The $J_{0,\ rad}$ was estimated by using a method same as the previous reports.[9,10] In brief, the black body irradiance is:

$$\emptyset_{BB} = \frac{1}{4\pi^2(\frac{h}{2\pi})^3 c^2} \times \frac{E^2}{expexp\left(\frac{E}{k_BT}\right) - 1} \qquad Equation\ S1$$

With h Plank's constant, k_B Boltzmann constant and T temperature. At room temperature, the dark radiative recombination current ($J_{0,rad}$) is:

$$J_{0,rad} = e\int EQE(E) \times \Phi_{BB}(E)dE \qquad Equation\ S2$$

With EQE the measured photovoltaic external quantum efficiency. The calculated results are shown in Figure S5, with the $J_{0,rad}$ value of $1.43 \pm 0.07 \times 10^{-20}$ A/m^2 and $1.62 \pm 0.07 \times 10^{-20}$ A/m^2 for $MAPbI_3$ and $MA_{1-x}Gua_xPbI_3$ based devices, respectively.

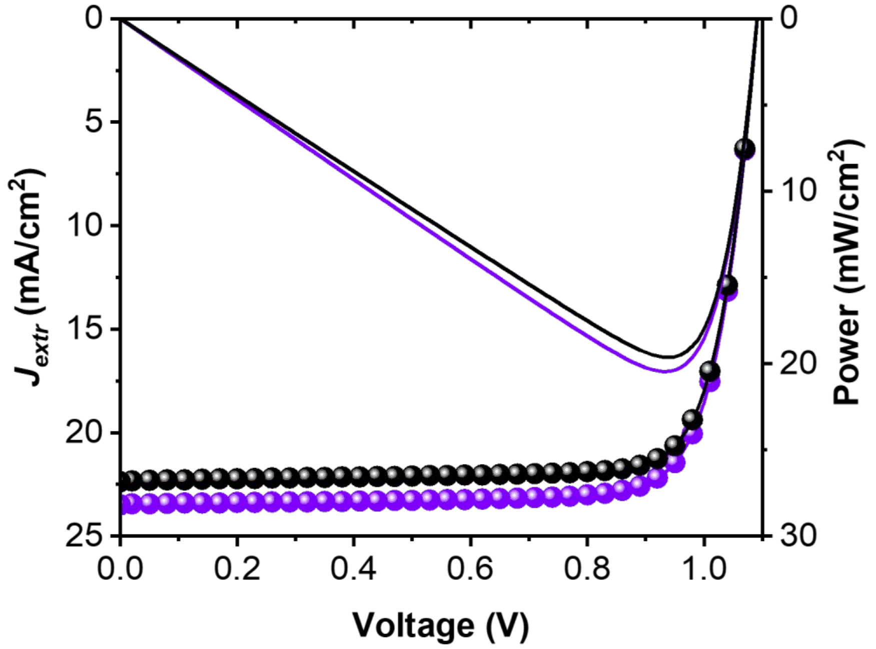

Figure S10. J-V and power characteristics of $MAPbI_3$ and $MA_{1-x}Gua_xPbI_3$ PSCs from operando PL measurement.

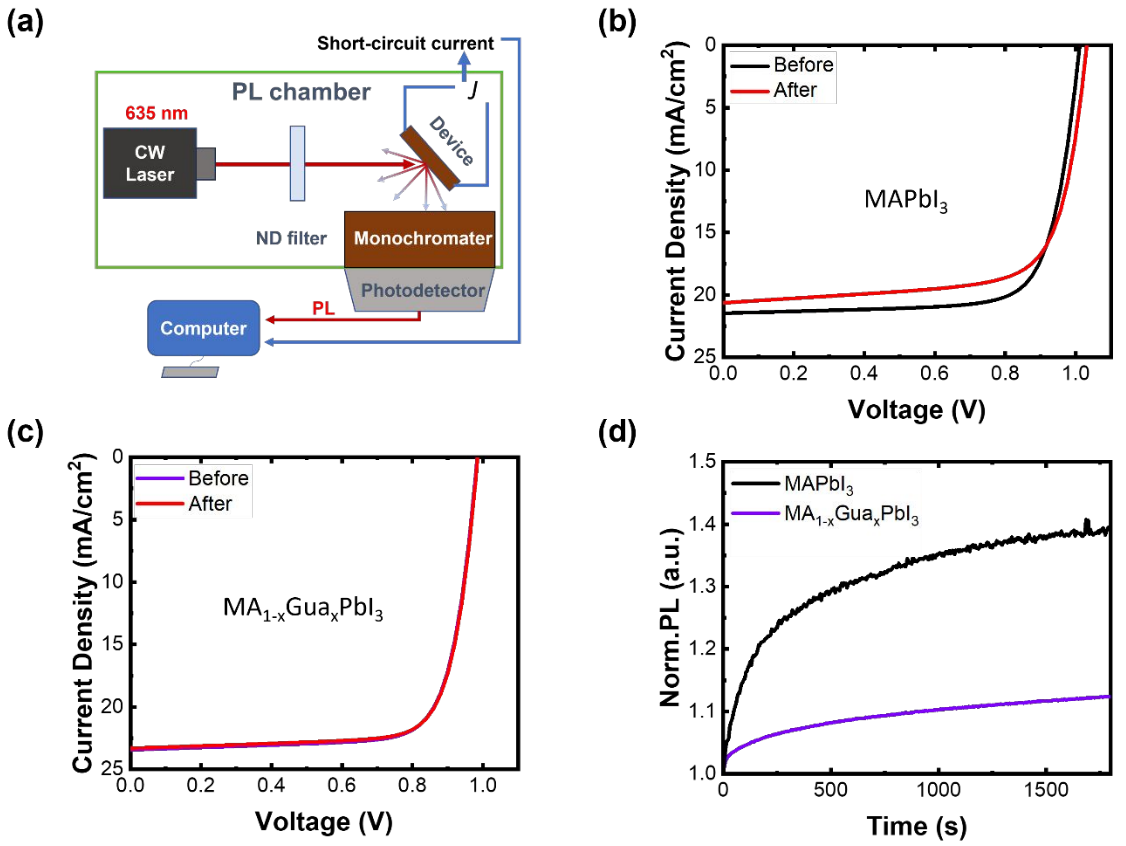

Figure S11. (a) Schematic drawing of the set-ups for operando time-dependent PL measurement. 1-sun-equivalent 635 nm CW laser illumination is used for excitation and PL intensity at wavelength of 780 nm was recorded. This ensured that the PL measurement conditions were comparable to real operational conditions. The intensity is calibrated by matching the short circuit current density under laser illumination to that under AM 1.5. (b-c) J-V curves of $MAPbI_3$ and $MA_{1-x}Gua_xPbI_3$ devices before and after operando time-dependent PL measurement at short-circuit (see Figure 3d). These curves were measured under AM 1.5G illumination by taking the devices out of the PL measurement chamber to a standard solar simulator. (d)Time-dependent PL measurement results of perovskite films on glass substrate using the same set-up and measurement condition as (a).

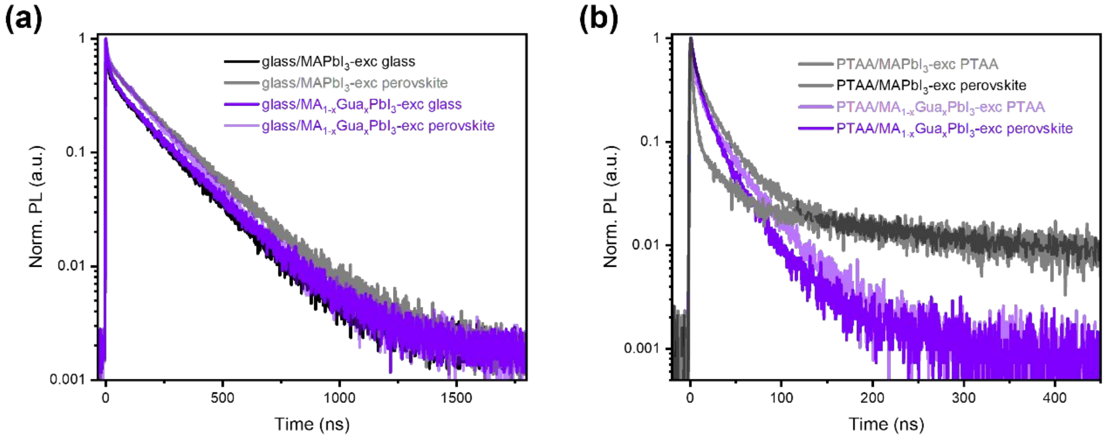

Figure S12. TRPL decay kinetics of (a) glass/perovskite and (b) ITO/PTAA/perovskite samples with front and back excitation using a 405 nm laser at a fluence of 10 nJ/cm² per pulse and a frequency of 500 kHz for (a) and 2 MHz for (b).

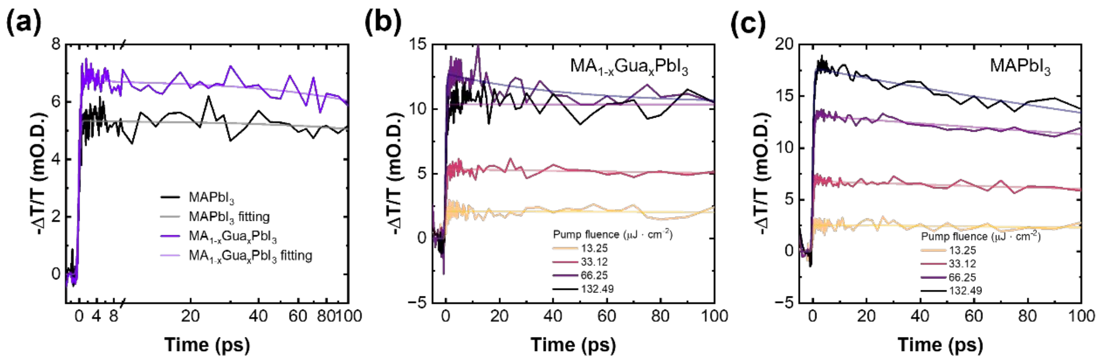

Figure S13. All optical-pump THz-probe dynamics are fitted using exponentials convoluted with a Gaussian function. (a) Optical-pump THz-probe dynamics of $MAPbI_3$ and $MA_{1-x}Gua_xPbI_3$ films on quartz substrates, with an excitation wavelength of 400 nm at 33.1 μJ·cm^{-2}. (b-c) Pump fluence dependence of optical-pump THz-probe dynamics for the $MA_{1-x}Gua_xPbI_3$ and $MAPbI_3$ films. At a pump fluence of 132.5 μJ·cm^{-2}, higher-order effects, such as the Auger effect, manifest.

References

(1) Milot, R. L.; Eperon, G. E.; Snaith, H. J.; Johnston, M. B.; Herz, L. M. Temperature-Dependent Charge-Carrier Dynamics in CH3NH3PbI3 Perovskite Thin Films. *Adv Funct Mater* **2015**, *25* (39), 6218–6227. https://doi.org/https://doi.org/10.1002/adfm.201502340.

(2) Kosasih, F. U.; Cacovich, S.; Divitini, G.; Ducati, C. Nanometric Chemical Analysis of Beam-Sensitive Materials: A Case Study of STEM-EDX on Perovskite Solar Cells. *Small Methods* **2021**, *5* (2), 2000835. https://doi.org/https://doi.org/10.1002/smtd.202000835.

(3) de la Peña, F.; Prestat, E.; Lähnemann, J.; Fauske, V. T.; Burdet, P.; Jokubauskas, P.; Furnival, T.; Francis, C.; Nord, M.; Ostasevicius, T.; MacArthur, K. E.; Johnstone, D. N.; Sarahan, M.; Taillon, J.; Aarholt, T.; pquinn-dls; Migunov, V.; Eljarrat, A.; Caron, J.; Nemoto, T.; Poon, T.; Mazzucco, S.; actions-user; sivborg; Tappy, N.; Cautaerts, N.; Somnath, S.; Slater, T.; Walls, M.; pietsjoh. Hyperspy/Hyperspy: V2.2.0. *Zenodo* **2024**, 4057415. https://doi.org/10.5281/zenodo.14057415.

(4) Pearson, K. LIII. On Lines and Planes of Closest Fit to Systems of Points in Space. *The London, Edinburgh, and Dublin Philosophical Magazine and Journal of Science* **1901**, *2* (11), 559–572. https://doi.org/10.1080/14786440109462720.

(5) Kosasih, F. U.; Su, F.; Du, T.; Ratnasingham, S. R.; Briscoe, J.; Ducati, C. Deep Learning-Assisted Multivariate Analysis for Nanoscale Characterization of Heterogeneous Beam-Sensitive Materials. *Microscopy and Microanalysis* **2023**, *29* (3), 1047–1061. https://doi.org/10.1093/micmic/ozad033.

(6) Cliff, G.; Lorimer, G. W. The Quantitative Analysis of Thin Specimens. *J Microsc* **1975**, *103* (2), 203–207. https://doi.org/https://doi.org/10.1111/j.1365-2818.1975.tb03895.x.

(7) Pawley, G. S. Unit-Cell Refinement from Powder Diffraction Scans. *J Appl Crystallogr* **1981**, *14* (6), 357–361. https://doi.org/10.1107/S0021889881009618.

(8) Rau, U. Reciprocity Relation between Photovoltaic Quantum Efficiency and Electroluminescent Emission of Solar Cells. *Phys Rev B* **2007**, *76* (8), 85303. https://doi.org/10.1103/PhysRevB.76.085303.

(9) Xu, W.; Hart, L. J. F.; Moss, B.; Caprioglio, P.; Macdonald, T. J.; Furlan, F.; Panidi, J.; Oliver, R. D. J.; Pacalaj, R. A.; Heeney, M.; Gasparini, N.; Snaith, H. J.; Barnes, P. R. F.; Durrant, J. R. Impact of Interface Energetic Alignment and Mobile Ions on Charge Carrier Accumulation and Extraction in P-i-n Perovskite Solar Cells. *Adv Energy Mater* **2023**, *13* (36), 2301102. https://doi.org/https://doi.org/10.1002/aenm.202301102.

(10) Caprioglio, P.; Stolterfoht, M.; Wolff, C. M.; Unold, T.; Rech, B.; Albrecht, S.; Neher, D. On the Relation between the Open-Circuit Voltage and Quasi-Fermi Level Splitting in Efficient Perovskite Solar Cells. *Adv Energy Mater* **2019**, *9* (33), 1901631. https://doi.org/https://doi.org/10.1002/aenm.201901631.
